# Patient‐specific finite element models of the human mandible: Lack of consensus on current set‐ups

**DOI:** 10.1111/odi.13381

**Published:** 2020-07-09

**Authors:** Bram Barteld Jan Merema, Joep Kraeima, Haye H. Glas, Fred K. L. Spijkervet, Max J. H. Witjes

**Affiliations:** ^1^ Department of Oral and Maxillofacial Surgery University Medical Center Groningen Groningen The Netherlands

**Keywords:** CAD‐CAM, finite element analysis, in vitro validation, mandibular reconstruction, patient‐specific modelling, prosthesis and implants

## Abstract

The use of finite element analysis (FEA) has increased rapidly over the last decennia and has become a popular tool to design implants, osteosynthesis plates and prostheses. With increasing computer capacity and the availability of software applications, it has become easier to employ the FEA. However, there seems to be no consensus on the input variables that should be applied to representative FEA models of the human mandible. This review aims to find a consensus on how to define the representative input factors for a FEA model of the human mandible. A literature search carried out in the PubMed and Embase database resulted in 137 matches. Seven papers were included in this current study. Within the search results, only a few FEA models had been validated. The material properties and FEA approaches varied considerably, and the available validations are not strong enough for a general consensus. Further validations are required, preferably using the same measuring workflow to obtain insight into the broad array of mandibular variations. A lot of work is still required to establish validated FEA settings and to prevent assumptions when it comes to FEA applications.

## 1 INTRODUCTION

Over the last decennia, a large variety of osteosynthesis plates and prostheses has been presented in the literature for the treatment of, for example, trauma, oncology or TMJ patients. These applications have in common that failure (loosening or fracturing) of the implanted material, or the screw‐fixation of the implant to the patient's bone will often result in failure of the application. It appears crucial for success that the implanted osteosynthesis plates and its fixations are of a matching strength for the patient's specific situation. Mandibular reconstruction failure through either osteosynthesis plate failure or screw loosening are widely reported in the literature as the most common causes of mechanical failure(Freitag, Hell, & Fischer, 1991; Irish et al., 1995; Kimura et al., 2006; Markwardt, Pfeifer, Eckelt, & Reitemeier, 2007; van Gemert et al., 2012), which shows the lack of a truly universal reconstruction solution due to the uniqueness of each patient's reconstruction situation. The accessibility and pricing of confection sized and shaped osteosynthesis plates have resulted in them being commonly adopted worldwide. These plates, however, are associated with the reported osteosynthesis failure in mandibular reconstructions (Maurer, Eckert, Kriwalsky, & Schubert, 2010; Schoning & Emshoff, 1998; Shibahara, Noma, Furuya, & Takaki, 2002). There is seemingly a sophisticated balance between plates being too weak, causing material failure, or too strong, potentially leading to stress‐shielding or disturbance of the natural equilibrium of bone formation, causing resorption of the surrounding bone (Kennady, Tucker, Lester, & Buckley, 1989). The development of biomechanical models to describe the forces acting on the mandible, using specifically the finite element analysis (FEA), has been underway over the last decennia in for example automotive or aviation engineering. Accurate FEA models help to predict material behaviour without the need for destructive tests and could replace in vivo tests. With the increase in computer capacity and the availability of software applications, FEA has gained ground in the biomedical field since the 1970s and has proven valuable due to its non‐destructive character and ease in evaluating multiple scenarios (Huiskes & Chao, 1983).

**FIGURE 1:**
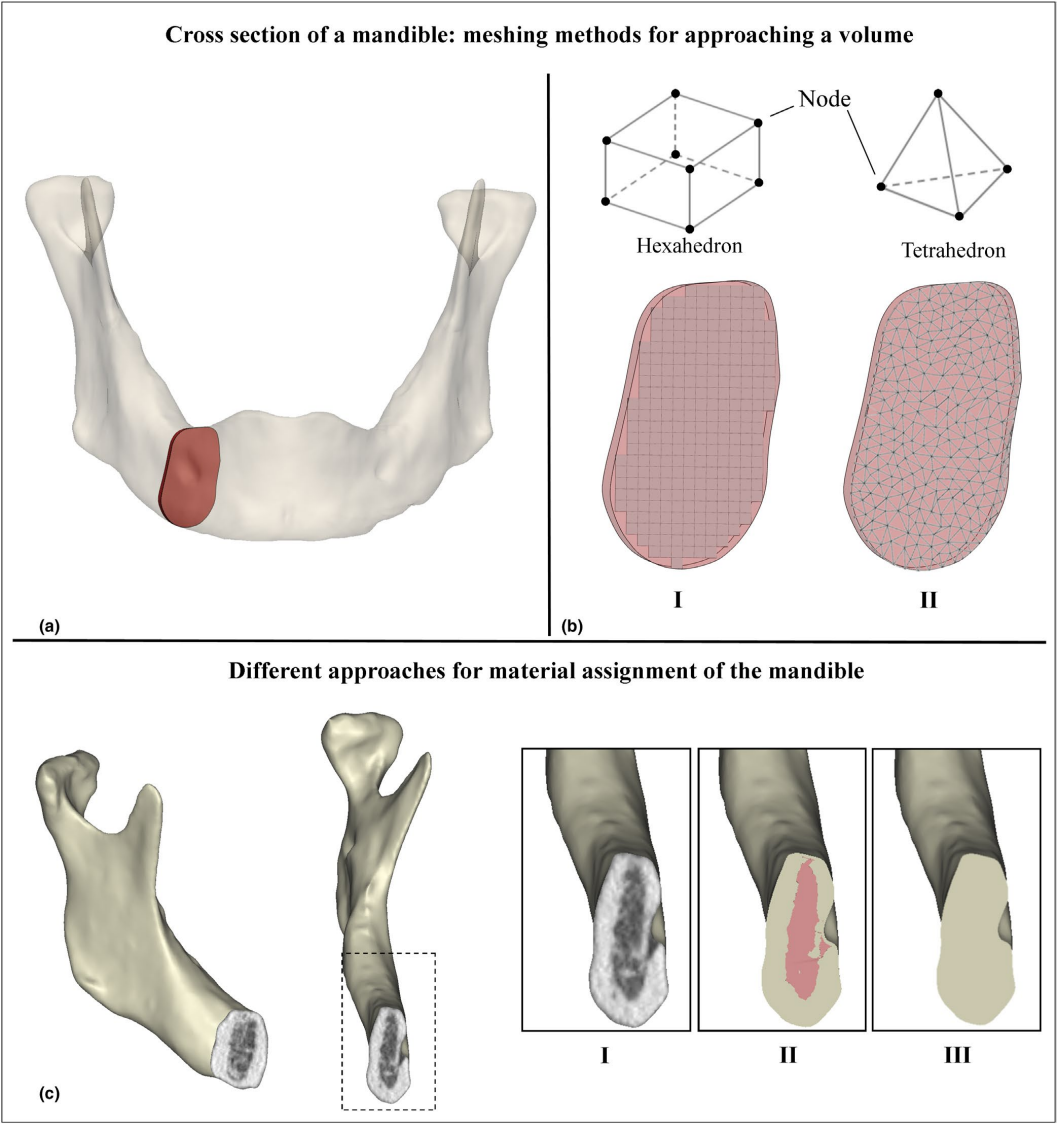
(a) Indication of the position of the red coloured slice, used in (b) to illustrate the approximation of the shape of this slice using hexahedal (b‐I) and tetrahedral (b‐II) meshes with the same dimensions. The number of nodes is highly influenced by this and this is reflected in the outcome of the FEA. (c) A 3D model of a mandible showing the CT pixels with material information on the cut planes. Approach c‐I represents material assignment per voxel, approach c‐II shows the assumption of two different materials (cortical and cancellous) and approach c‐III illustrates a solid uniform (cortical only) material assignment of the mandible. These settings affect the outcome of the FEA

There are great variations between the FEA models due to differences in the input factors such as constraints, load application, mechanical properties of the bone, muscle forces and muscle force directions. Xin et al. (2013) describe three different material composition options to approach the mandible mechanically, namely a solid model with homogeneous material properties (Kavanagh et al., 2008) and two solid models composed of cortical and cancellous volume, each with their own homogeneous material properties (Gautam, Zhao, & Patel, 2011; Xiangdong, Limin, & Shizhen, 2012) and a heterogeneous or orthotropic material assignment, meaning the bulk material properties are not the same in all directions (Bujtar, Simonovics, Varadi, Sandor, & Avery, 2014; Huang, Tsai, Lin, Chien, & Hsu, 2010; Taylor et al., 2002) (Figure 1). Generally, loads are approached with simplified musculatory models, of only one muscle group or resultant force. Other studies have created a more extensive and complex model consisting of four or more muscle groups (Commisso, Martinez‐Reina, Ojeda, & Mayo, 2015; Korioth & Hannam, 1994; Vajgel et al., 2013). In the latter, the masseter (deep and superficial or combined), temporalis (anterior and medial or combined), lateral pterygoid and medial pterygoid are typically defined. The force magnitudes and working directions are, however, not commonly agreed upon and vary considerably (Koolstra & van Eijden, 1999, 2001; Koolstra, van Eijden, Weijs, & Naeije, 1988; May, Saha, & Saltzman, 2001; Meyer, Kahn, Boutemy, & Wilk, 1998). Muscle forces and directions are subject‐specific; yet, most authors describe a universal and simplified musculatory model which should represent a maximum loading. In static engineering, this might seem a safe solution to a structural problem; but, for a mandible, this could result in unnecessarily strong and bulky plates which do not fit the specific patient (e.g. a small, resorbed mandible due to an edentulous situation does not allow placement of a bulky implant).

Current literature lacks consensus on the input variables that should be applied to representative FEA models of the human mandible. Thus, the aim of this review was to find a consensus on how to define the input factors for a representative FEA model of the human mandible.

## 2 METHODS

A computer database search was carried out in PubMed on the 1 November 2019. The applied search term was created using both MeSH and Boolean terms: *(“Mandible”[Mesh]* ***OR*** *mandible[tiab]* ***OR*** jaw*[tiab]) ***AND*** (finite element*[tiab] ***OR*** *fea[tiab])* ***AND*** *(“Laboratory”[Mesh]* ***OR*** *“*In Vitro*Techniques”[Mesh]* ***OR*** *“Validation Studies as Topic”[Mesh]* ***OR*** validation*[tiab] ***OR*** in vitro*[tiab]* ***OR*** in‐vitro*[tiab]* ***OR*** *test[tiab]* ***OR*** *assess[tiab]* ***OR*** *verification[tiab])* ***NOT*** *(dental* ***AND*** *Humans[Mesh])*. Subsequently, a search was performed in the Embase database using the same separated terms.

The resulting abstracts, or entire content when the abstract did not provide sufficient information for inclusion or exclusion, were assessed by one author (BJM). No criterion was set regarding the date of publication. The applied criteria were as follows:

### 2.1 Inclusion criteria

1. Written in the English language
2. Assessment of one or more human mandible(s); only human models were taken into account in order to make comparison of multiple studies possible.
3. In vitro model with matching finite element model; the study should compare in vitro mechanical testing with a FEA model of the matching mandible(s).

### 2.2 Exclusion criteria

1. Use of synthetic or phantom mandibles**;** the aim of this study was to extract representative material properties for the mandible. Synthetic bone substitutes introduce assumptions and confounders to the models.
2. Focus on soft tissue; our study focuses on hard tissue as most FEA models are used to design osteosynthesis materials or implants.

### 2.3 Rationale

A FEA model is typically composed of an object, referred to as a geometry, to which material properties, boundary conditions and loads are assigned. Material characteristics, such as yield properties, elastic behaviour or Young's Modulus and Poisson's ratio, are important input parameters and should be considered carefully in order to obtain a representative reflection of the anatomical situation. The Young modulus (YM), expressed as E [GPa], measures elasticity; the higher the YM the stiffer the material becomes. The Poisson ratio (PR) [dimensionless] of a material describes the deformation behaviour under a load and is calculated by dividing the amount of transversal expansion by the amount of axial compression. Also, relevant to a FEA are yield properties. These indicate a stress value where a material will start to yield and, in order to avoid this, the minimal value must be known.

Boundary conditions relate to the constraints that are applied to the geometry, in this case the mandible. When it comes to a FEA of the mandible, fixation in an area of the mandible at, for example, both condyles, and a limitation in the freedom of movement at the occlusal site are typical examples of boundary conditions. Another type of boundary condition that often needs to be applied in a FEA is a contact set. Contact sets are required when multiple geometries or parts of a geometry are in contact and they define how the FEA software should treat the contacting sites. For example a mandible that is considered to consist of a cortical shell with a cancellous inner volume (Figure 1c‐II). If both materials have different properties, these will have the tendency to deform differently at the sites of contact. During a FEA, the user has to decide whether to treat the two materials as fixed, thus, prohibiting interbody movement, or to apply a bearing contact set and allowing for shear movement at the contact site.

Mesh creation (meshing) is the discretisation step of FEA in which the analysed object is described as a finite number of blocks or elements (Figure 1a,b). This step is responsible for the division of the greater numerical problem into a finite number of smaller problems and is of great importance since the mesh parameterisation can drastically influence the approximation of the input geometry and thus the quality of the FEA's results (Ramos & Simoes, 2006).

Forces are generally applied by assigning loads, or loading conditions. In order to assign a load, a force origin and direction are required alongside the force magnitude. The mandible's loading conditions, representing the musculatory system, could be approached in this manner.

When performing a FEA, it is important to pay attention to any occurring stress and strain. In order to understand whether or not the results are within acceptable limits, the limitations of the acceptable magnitudes of stress and strain should be clear. These limitations have been known for years for the vast majority of engineering materials but appear to be complex for a natural and dynamic material such as bone due to its heterogeneous and individual character (Dechow, Nail, Schwartz‐Dabney, & Ashman, 1993; Keller, 1994; Rho, Hobatho, & Ashman, 1995). For FEA, it would be interesting to know what yield strength and fatigue strength should be taken into account.

In accordance with the above, six main categories were formulated and used to assess all the included papers:

- Bone geometry and property acquisition
- Acceptable bone stress values
- Musculatory model and fixtures
- Applied finite element settings
- In vitro validation method
- In vitro and FEA results conformity

## 3 RESULTS

The PubMed electronic database search resulted in a total of 137 papers matching our search term, published between 1992 and 2019. Seven papers complied with the inclusion criteria, as shown in Table 1 (Clason, Hinz, & Schieferstein, 2004; Gröning, Liu, Fagan, & O'Higgins, 2009; Mesnard & Ramos, 2016; Ramos, Gonzalez‐Perez, Infante‐Cossio, & Mesnard, 2019; Ramos & Mesnard, 2016; Ramos, Nyashin, & Mesnard, 2017; Vollmer, Meyer, Joos, Vègh, & Piffkò, 2000; Xin et al., 2013). Of these, four papers were written by the same authors (Ramos & Mesnard) (Mesnard & Ramos, 2016; Ramos et al., 2017, 2019; Ramos & Mesnard, 2016) who used of the same or a very similar FEA model and validation method, resulting in a total of four unique study models to compare. The Embase search that followed the PubMed search did not result in any additional further unique studies.

**TABLE 1:** The finite element and in vitro approaches of all the included papers

Author	FEA‐model approach	Cortical		Cancellous		In vitro method	Conformity FEA & in vitro results
		*E* (GPa)	*ν* (−)	*E* (GPa)	*ν* (−)		
Vollmer et al. (2000)	Homogeneous, cortical and cancellous	NS	NS	NS	NS	Load applying apparatus and strain gauge measurement	0.992 correlation coefficient
Clason et al. (2004)	Homogeneous, cortical and cancellous	Dry 5.466	0.246	0.646	0.246	Load applying apparatus and optical measurements method	Average relative mean square deviations of the marker positions: 0.29, 0.28 and 0.29 mm for 3 load cases
		Soaked 5.658	0.273	0.785	0.269		
Gröning et al. (2009)	Homogeneous, cortical	17	0.3	NA	NA	Load applying apparatus and Digital Speckle Pattern Interferometry measurement	Most predicted values lie within 2 *SD*
Ramos and Mesnard (2016)	Homogeneous, cortical and cancellous	14.7	0.3	0.4	0.35	Load applying apparatus and strain gauge measurement	*R* ^2^: .931, slope: 1.05, NRMSD: 10.4%
Mesnard and Ramos (2016)	Homogeneous, cortical and cancellous	14.7	NS	0.4	NS	Load applying apparatus and strain gauge measurement	*R* ^2^: .935, slope: 1.045, NRMSD: 10.3%
Ramos et al. (2017)	Homogeneous, cortical and cancellous	13.7	0.3	0.4–13.7	0.3	Load applying apparatus and strain gauge measurement	*R* ^2^ slope: 0.953, NRMSD: 9.7%
Ramos et al. (2019)	Homogeneous, cortical and cancellous	14.7	0.3	0.4	0.35	Load applying apparatus and strain gauge measurement	*R* ^2^: .95, slope: 0.899, NRMSD: 6.5%

Note

*E*, Young's modulus; GPa, 1*10^9^ Pascal; NA, not‐applicable; NRMSD, normalised root‐mean‐square deviation; NS, not specified; *R* ^2^, correlation value; *SD*, standard deviation; *ν*, Poisson's ratio.

### 3.1 Bone geometry and property acquisition

The mandibular geometry in all the seven papers included in this study was obtained through segmentation of computed tomography (CT) or cone beam CT (CBCT) imaging. Ramos and Mesnard chose to perform a micro‐CT scan of the mandibles (Mesnard & Ramos, 2016; Ramos & Mesnard, 2016; Ramos et al., 2017, 2019), whereas all other authors used regular CT scans (Clason et al., 2004; Vollmer et al., 2000). Gröning et al. (2009) performed both a micro‐CT and a regular CT and created a high‐resolution and lower resolution model with element counts and sizes of 19.6 million and 0.135 mm/450.000 and 0.488 mm, respectively. They found that both their models predicted similar strains and noted that a relatively low‐resolution scan is sufficient for FEA‐model creation. However, the resolution of the scan must be increased when assessing strain gradients in small structures. Thus, a regular (CB)CT could be sufficient for modelling a human mandible in toto when the region of interest of the mandible is not at micro level.

The (CB)CT data (DICOM files) possess information on both geometry and local radiographic attenuation. Local density values can be assigned mathematically to the latter. Through mathematical formulae, the attenuation values, expressed in Hounsfield unit (HU) or grey value (GV), and material properties such as YM and PR can be extracted. The formulae used in the literature for this extraction were empirically determined and differ from each other since the tested samples origin from different anatomical positions (Cioffi et al., 2007; van Ruijven, Mulder, & van Eijden, 2007; Xin et al., 2013).

All the seven papers included in this study describe an in silico model where the material properties are considered homogeneous(Clason et al., 2004; Gröning et al., 2009; Mesnard & Ramos, 2016; Ramos & Mesnard, 2016; Ramos et al., 2017, 2019; Vollmer et al., 2000). Clason et al., Ramos and Mesnard, Mesnard and Ramos and Vollmer et al. assigned two material groups, related to HU, in conformation with the Ciarelli, Goldstein, Kuhn, Cody, and Brown (1991) study and created a cortical and a cancellous mandibular portion. Ramos et al. (2017) assumed the teeth are part of the cortical volume, stating this would have marginal influence on mandible behaviour. Gröning et al. (2009) left the cancellous portion out of their model and used a single pair of material properties for cortical bone instead. Their studied mandible was assumed to be fully cortical even though it was dentate. A relatively wide range of YM was applied to the homogeneous models. Clason et al. (2004) applied the most flexible value, namely 5.46 [GPa] while Gröning et al. (2009) used the stiffest value, 17 [GPa]. The PR of mandibular (cortical) bone was often chosen 0.3 (Borchers & Reichart, 1983; Gallas Torreira & Fernandez, 2004; Ichim, Swain, & Kieser, 2006; Korioth, Romilly, & Hannam, 1992; Liu, Fan, & Qian, 2008; Meijer, Kuiper, Starmans, & Bosman, 1992; Nagasao, Kobayashi, Tsuchiya, Kaneko, & Nakajima, 2003; Ozan & Ramoglu, 2015; Santos et al., 2015). The studies that mechanically tested the PR of the mandible in different directions showed values ranging from 0.18 to 0.53 (Dechow et al., 1993; Schwartz‐Dabney & Dechow, 2003). We find that five of the selected studies had a PR of 0.3 or close to it (Gröning et al., 2009; Mesnard & Ramos, 2016; Ramos & Mesnard, 2016; Ramos et al., 2017, 2019). Clason et al. (2004) deviated from this with their values of 0.25–0.27 for cortical bone and as much as 0.65–0.79 for cancellous bone. Clason et al. (2004) studied the mechanical properties of mandibular bone in an inverse manner. They performed measurements on a cadaveric mandible prior to setting up an in silico model and adjusted the mechanical property settings to fit the in vitro measurements. Ramos used the cortical and cancellous bone values (PR 0.3 and 0.35, respectively) in one paper but applied the value of 0.3 to both the cortical and cancellous bone in a later study (Ramos & Mesnard, 2016; Ramos et al., 2017). The effect of which is expected to be marginal.

### 3.2 Acceptable bone stress values

None of the included papers paid attention to a maximum acceptable bone stress, yield properties or fatigue limits and thus did not state maximum values.

### 3.3 Musculatory models

The description of the musculatory models used in the included studies was in most cases rather brief. In order to simplify their mathematical model, Clason et al. (2004) chose to only apply a pterygo‐masseter sling which looped around the mandibular anguli and pulled upwards. The force applied to this sling went up to 650 [N] which, according to Clason et al., covers the reasonable physiological range reported in the literature (Gay, Rendell, Majoureau, & Maloney, 1994; Kampe, Haraldson, Hannerz, & Carlsson, 1987). The simplification with such an approach makes comparison of the FEA model with the in vitro measurements easier, with less introduction of errors, while loading the mandible in a non‐physiological manner. Gröning et al. (2009) loaded their in vitro model by resting the mandible on both condyles and the lower incisors while vertically applying a force to the mandibular angles, which is comparable to Clason's et al.'s loading. Mesnard and Ramos (2016) and Ramos and Mesnard (2016) on the other hand did not add a physiologically complete musculatory model to their experiment but chose to apply a resultant force to the condyle since the condyle and mandibular ramus were their regions of interest. Vollmer et al. (2000) applied a load of 130 N to both coronoid processes to imitate mastication through the temporalis muscle only. Ramos et al. (2017) used the most extensive musculatory model. It involved five pairs of muscle forces, including all the previously studied vector directions (Mesnard et al., 2011). All the selected studies agree on the exclusion of the lateral pterygoid muscle from the analysis because this muscle is located at the condyle; all their mandibles were either fixed or loaded with a resultant TMJ force at the condyles.

### 3.4 Applied finite element settings

Clason et al. (2004) and Gröning et al. (2009) describe a vertical rigid fixation of the condyle surfaces, which was the direction of load application. Gröning et al. also fixed the tips of the anterior teeth, preventing movement in this direction. The highly similar models used by Mesnard & Ramos (2016) and Ramos and Mesnard (2016) focused on the mandibular ramus and condyle. The hemi‐mandible was fixed at the mandibular body, by cementation to the testing apparatus. Therefore, the applied fixture in the FEA was matched to the physical situation. Ramos et al. (2017) fixed a hemi‐mandible to his in vitro tool by cementation but created a FEA model which included a musculatory model and boundary conditions to the intact mandible. The condyles were fixed in the craniocaudal and anteroposterior direction while a lower incisive tooth was fixed in mediolateral and craniocaudal direction. Vollmer et al.'s (2000) description of the FEA‐model set‐up was very minimal. They did not give any specific details of the FEA fixation of the tested mandible other than that the condyles of the mechanically tested mandible were fixed and this was simulated in the FEA model.

Gröning et al. (2009) and Vollmer et al. (2000) used voxel(3D pixel)‐to‐voxel material assignment. That is, for every single voxel, or group of voxels in the CT data, one was created in the FEA mesh (Figure 1c‐I). This resulted in linear hexahedral elements with 8 connective corner points between elements, called nodes (Figure 1b‐I). Clason et al. (2004), Mesnard and Ramos (2016), Ramos and Mesnard (2016), and Ramos et al. (2017, 2019) and made use of linear tetrahedral elements which consist of 4 corner nodes and can be presented as a pyramid shape (Figure 1b‐II). Linear tetrahedral elements can be fit into complex geometries more accurately with relatively bigger element dimensions due to their pyramid shape. An organically shaped mandible for example would need a relatively high number of hexahedral elements in order to follow the outer surface accurately (Figure 1b). Moreover, wherever there are sharp angled boundaries between elements (sharp edges), it is likely the peak stresses will be concentrated. A typical linear hexahedral mesh shows these peak stress concentrations when the element's dimensions are too big.

Interbody contact sets are applied wherever there are multiple objects coinciding. Of the four authors that actually made use of multiple contacting bodies Clason et al. (2004), Mesnard and Ramos (2016), Ramos and Mesnard (2016), Ramos et al. (2017, 2019) and (Vollmer et al., 2000), only Mesnard and Ramos described the applied interbody contact for the cortical and cancellous volumes. They reported a “glue contact*”* which allows for interface separation. Furthermore, they mentioned a friction contact for the mandible‐implant and screw‐implant interfaces. Ramos and Mesnard (2016) described a comparable contact between the mandible and implant.

The software used for the FEA varied in the included papers from in‐house code (Clason et al., 2004) to non‐commercially available VOX‐FE software (Gröning et al., 2009) and commercially available software. Mesnard and Ramos (2016), Ramos and Mesnard (2016), and Ramos et al. (2017, 2019) used a separate preprocessor (HyperWorks 12; Altair Engineering) and performed their analysis in MSc MARC 2015 (MSc Software). Vollmer et al. (2000) chose to combine an in‐house preprocessor to mesh the CT data with a commercially available solver (Cosmos V2.0).

### 3.5 In vitro validation methods

In most included studies, in vitro measurements of the mandibles are carried out by strain gauges. Usually, a series of strain gauges is applied to the surface of the region of interest by means of an adhesive. When the studied object is subject to surface deformation, the strain gauges will change length and width thereby changing the electrical resistance, which can be measured. In Mesnard and Ramos (2016), Ramos and Mesnard (2016), Ramos et al. (2017, 2019) and Vollmer et al. (2000), method, a series of strain gauges were applied to the mandibular surface in the regions of interest and the measured values were mathematically converted into local strain values. In all cases, the in vitro loads were applied by means of a compression or tensile testing machine. Also, Gröning et al. (2009) used a tensile testing machine for the load applications but, instead of strain gauges, applied digital speckle pattern interferometry (DSPI) in order to optically perform their measurements.

Clason et al. (2004) describe an in vitro experiment that measured the displacement of specific points of interest on the mandible. A number of tracer spheres were applied to the mandibular surface and the displacement was recorded using a camera. Contrary to the afore‐mentioned studies, the loads were applied by a series of hydraulic actuators.

They preserved the mandible in alcohol and measurements were performed under dry conditions. Ramos and Mesnard also first used a cleaned fresh frozen mandible (Mesnard & Ramos, 2016; Ramos & Mesnard, 2016), but the teeth were removed. However, in their follow‐up study (Ramos et al., 2017), the teeth were left in the mandible. Vollmer et al. (2000) chose to store their five mandibles in a humid 20°C atmosphere by soaking the mandibles in a physiological sodium chloride solution one hour prior to mechanical testing.

### 3.6 In vitro and FEA results conformity

All the studies claim a good correlation between the FEA outcome and in vitro results. The results of the studies however are difficult to compare since the raw data are unavailable and most of the studies scored their results differently. The studies that present a correlation coefficient show good correlations of 0.992 ((Vollmer et al., 2000), 0.931 (Ramos & Mesnard, 2016), 0.935 (Mesnard & Ramos, 2016), 0.953 (Ramos et al., 2017) and 0.95 (Ramos et al., 2019)). Most of the FEA results obtained by Gröning et al. (2009) lie within two standard deviations from the in vitro values. The results by Clason et al. (2004) are expressed as average relative mean square errors between the predicted and measured displacement. The calculated errors were 0.29 mm, 0.28 mm and 0.29 mm for their first, second and third load cases, respectively.

## 4 DISCUSSION

Finite element studies are currently recognised as a necessary part of designing personalised osteosynthesis. However, the approaches vary greatly and the assumptions, concerning for example material properties and boundary conditions, are not fully understood. We aimed to find a consensus on how to approach a representative FEA model of the human mandible within the literature.

Many studies used FEA to develop and design osteosynthesis and prostheses for patients but surprisingly, only seven papers describe how they validated the applied FEA on human mandibles. This simulation technique supplies the user with results that match the input problem as formulated by the user, even if this input problem is not an accurate one. Thus, the reader should be critical towards the used approach.

The papers included in this current study applied similar simplifications regarding bone geometry and material properties. They all describe homogeneous materials with either only a cortical portion or a cortical portion with cancellous volume. The properties required for an FEA, that is YM and PR, were mentioned by every study and they generally agreed with prior experiments (Dechow et al., 1993; Schwartz‐Dabney & Dechow, 2003). The majority of reported PR vary marginally and ranged from 0.25–0.30 to 0.25–0.35 for cortical and cancellous bone, respectively. Clason et al. (2004) calculated PR and YM, however, both seem to stand out (Table 1). However, the majority of the YM ranged from 13.7 to 17 [GPa], Clason et al. calculated values of 5.5–5.7 [GPa] for the cortical bone, resulting in a much stiffer material (Ramos et al., 2017). This study involved only one test subject and the diverging results could have been caused by individual variables (age, porosity or geometry, etc.), or measuring variables. For such outlying results, we find the support too weak to use their measured material properties over the properties used in the rest of the included studies.

Xin et al. (2013) applied heterogeneous material properties whereby the material property assignment was matched to the local attenuation properties. They divided the CT GV range into ten equally distributed intervals and assigned a set of material properties to each of these intervals, creating ten different materials. They describe in vitro measurements of mandibular segments in three different directions. Their results show property independency in all the measured directions and specimen locations. Hart, Hennebel, Thongpreda, Van Buskirk, and Anderson (1992), however, describe that even on taking the anisotropy of the mandible into account, two out of three directions show similar properties. Schwartz‐Dabney and Dechow (2003) show the varying properties of cortical bone related to the location in the mandible. This method should be applied patient‐specifically due to the great individuality of the mandibular shape and bone quality. However, validations of this method are still lacking.

The focus of the seven included papers was clearly not on the application of a representative musculatory model in either the FEA or in vitro experiments. Except for Ramos et al. (2017), who describe five muscle groups per side, all the authors simplified their model to one force. This might be sufficient when analysing only a part of the mandible but the lack of use of the entire mandible should be acknowledged. Only applying a resultant force, calculated for a specific region, could result in overlooking the internal force transmission. All the authors agree on the elimination of the lateral pterygoid muscle from the analysis and using the condyles to either fixate the mandible in space (Clason et al., 2004; Gröning et al., 2009; Vollmer et al., 2000), or to apply a resultant force (Mesnard & Ramos, 2016; Ramos & Mesnard, 2016; Ramos et al., 2017, 2019).

Most of the FEA‐related publications do not describe the maximum bone performance values in terms of yield or fatigue properties. Since bone is a dynamic material, subject to a number of factors influencing its properties and with a continuous (de)formation, it is imaginable that it is not possible to measure true in vivo properties such as fatigue in vitro. Zioupos & Casinos (1998) studied fatigue damage in cadaveric femoral bone in an in vitro setting and show the influence of order in which non‐uniform repetitive loadings are applied, indicating simple stress against cycles to failure (S‐N) fatigue tests do not suffice for ex vivo bone. They conclude it is an extremely difficult task to predict in vivo bone fatigue under variable loading. Yield information can be obtained but is strongly dependant on the assumed material YM and PR. We found only two studies that state the yield strength and/or ultimate strength of the cortical and cancellous bone (Chen et al., 2018; Hoefert & Taier, 2018). These values, however, vary considerably. A third study extracted these ultimate values from experiments conducted with vertebral and femoral specimens and used this to calculate the values for the mandible (Kharmanda & Kharma, 2017). Of the seven included papers, only Mesnard and Ramos (2016), Ramos and Mesnard (2016), and Ramos et al. (2017, 2019) refer to fatigue, and relate this to strain instead of stress. The same strain focus was applied in the Bujtar et al. (2014) and Chen et al. (2018) studies which are related to Frost's “Mechanostat” principle (Frost, 2003), a principle in which bone formation and resorption are linked to the bone's strain values.

In vitro testing of mandibles can be approached with different measurement techniques, all with their own strengths and limitations. The optical techniques are “non‐invasive” and do not require the attachment of materials to the bone but are sensitive to vibration and light and require high‐resolution optical cameras. The use of gauges requires bone preparation, that is fixation of the sensor, and covers only one‐directional measurements. It should be mentioned that both techniques are only capable of surface measurements. Mesnard and Ramos (2016), Ramos and Mesnard (2016), and Ramos et al. (2017, 2019) could reproduce their FEA measurements in vitro multiple times. It is valuable that four papers included in this study applied the same in vitro method and calculated comparable and good regression values with their strain gauge workflow.

The small number of inclusions is the biggest limitation of this current study. The information available regarding validated FEA of the human mandible is scarce and that is worrying knowing how widely FEA are performed and used for the design of medical devices. FEA models could be more individual in order for patient‐specific plates and prostheses to better fit the patient in terms of bulkiness and in situ performance. A technique that should be considered is topology optimisation (TO). TO is a mathematical method applied in the FEA phase and is capable of removing material that dependant to the input variables, is unnecessary (Iqbal et al., 2019; Sutradhar et al., 2016). Instead of testing a man‐made design with FEA, we can have the TO calculate the ideal design by removing material from a volume, given certain boundary conditions.

In conclusion, we carried out a literature search to find a possible consensus on how to perform a FEA on the human mandible. The available validations provide a lot of information but appear insufficient for reaching a general consensus. Further validations are required, preferably using the same measuring workflow and multiple mandibles to obtain insight into the broad range of mandibular characteristics. We believe the models suggested by Mesnard and Ramos (2016), Ramos and Mesnard (2016), and Ramos et al. (2017, 2019) over the years are the most complete and best validated.

## CONFLICT OF INTERESTS

None.

## AUTHOR CONTRIBUTION

**Bram Barteld Jan Merema:** Conceptualization; Formal analysis; Software; Validation; Writing‐original draft. **Joep Kraeima:** Conceptualization; Formal analysis; Writing‐review & editing. **Haye H. Glas:** Conceptualization; Methodology; Writing‐review & editing. **Fred K. L. Spijkervet:** Conceptualization; Methodology; Supervision; Writing‐review & editing. **Max Witjes:** Conceptualization; Methodology; Supervision; Writing‐review & editing.
